# Organizing mechanism-related information on chemical interactions using a framework based on the aggregate exposure and adverse outcome pathways

**DOI:** 10.1016/j.envint.2020.105673

**Published:** 2020-03-24

**Authors:** Paul S. Price, Annie M. Jarabek, Lyle D. Burgoon

**Affiliations:** a Center for Public Health and Environmental Assessment, Office of Research and Development, U.S. Environmental Protection Agency, 109 TW Alexander Drive, Research Triangle Park, NC 27711, United States; b Environmental Laboratory, US Army Engineer Research and Development Center, Research Triangle Park, NC, United States

**Keywords:** Chemical interactions, Mixture toxicity, Adverse outcome pathway, Aggregate exposure pathway

## Abstract

This paper presents a framework for organizing and accessing mechanistic data on chemical interactions. The framework is designed to support the assessment of risks from combined chemical exposures. The framework covers interactions between chemicals that occur over the entire source-to-outcome continuum including interactions that are studied in the fields of chemical transport, environmental fate, exposure assessment, dosimetry, and individual and population-based adverse outcomes. The framework proposes to organize data using a semantic triple of a chemical (subject), has impact (predicate), and a causal event on the source-to-outcome continuum of a second chemical (object). The location of the causal event on the source-to-outcome continuum and the nature of the impact are used as the basis for a taxonomy of interactions. The approach also builds on concepts from the Aggregate Exposure Pathway (AEP) and Adverse Outcome Pathway (AOP). The framework proposes the linking of AEPs of multiple chemicals and the AOP networks relevant to those chemicals to form AEP-AOP networks that describe chemical interactions that cannot be characterized using AOP networks alone. Such AEP-AOP networks will aid the construction of workflows for both experimental design and the systematic review or evaluation performed in risk assessments. Finally, the framework is used to link the constructs of existing component-based approaches for mixture toxicology to specific categories in the interaction taxonomy.

## 1. Introduction

Toxicology, exposure science, and chemical risk assessment are in the midst of a transformation. Assessors are moving towards the use of *in vitro* assays and *in silico* predictions that provide insights on the mechanisms that cause adverse outcomes (AOs) (NRC, 2007). The methodologies driving this transformation have been referred to as New Approach Methodologies or NAMs (Pham et al., 2019; Wambaugh et al., 2019). *In vivo* toxicity data are limited to a relatively small number of substances. Because of the large, and increasing, number of chemicals in commerce it is envisioned that the majority of chemicals will be evaluated in the future using NAMs rather than data from *in vivo* models of toxicity (Kavlock et al., 2018). The benefits of NAMs are perhaps more critical to the study of the effects of chemical mixtures than the effects of single chemicals (Hernandez et al., 2019). There are more combinations of chemicals than individual chemicals and dose response for combined exposures are more complex than those for individual chemicals. Following Nelms et al. (2018) and Bopp et al. (2019), the term “chemical mixtures” is defined in this paper as an organism’s or population’s combined exposures to two or more chemicals, where the period of time between the exposures is sufficiently small as to allow the effects of one chemical to influence the response of the organism or population to one or more other chemicals. Chemical mixtures include intentional discrete mixtures (e.g., consumer products) and unintentional discrete mixtures (e.g., industrial effluents), and concurrent exposures to chemicals from multiple sources.

The hallmark of NAMs is to illuminate the mechanisms that determine the causal events in the source – exposure – dose – outcome continuum that describes the ability of a chemical to pose risks to humans and the environment (Cohen-Hubal et al., 2010; Hines et al., 2019). Data from *in vivo* and *in vitro* assays of toxicity and studies of metabolism and environmental fate are being collected, curated, and organized into databases (Thomas et al., 2019). *In silico* models based on the data are being used to predict the relationship between chemical structure and: biological activity and the ability to cause AOs (Patlewicz and Fitzpatrick, 2016; Patlewicz et al., 2018); the absorption and distribution characteristics of chemicals in biological systems (ten Berge, 2009; O’Connor et al., 2013; Sun et al., 2018); the physical and chemical properties of chemicals (Mansouri, 2018); and the functional roles of chemicals in commercial products (Phillips et al., 2017). Exposure-relevant data are being collected on the release of chemicals to the environment (Cashman et al., 2016; Smith et al., 2017); measurements of chemicals in environment, indoor dust, and biomonitoring samples (Sobus et al., 2018), and composition of consumer products (Dionisio et al., 2018). The prediction of internal doses and how they vary across individuals are being made for large numbers of chemicals using pharmacokinetic models (Ring et al. 2017; Pearce et al. 2017). Combined exposures to chemicals in consumer products are being modeled using databases of product-use patterns (Safford et al. 2017; Dudzina et al., 2015).

The large data sets generated by NAMs require frameworks to organize, hold, and facilitate their use in risk assessments. Two frameworks currently in use are the Adverse Outcome Pathway or AOP (Ankley et al. 2010; Ankley and Edwards, 2018) and the Aggregate Exposure Pathway or AEP (Teeguarden et al., 2016; Tan et al. 2018a; Tan et al., 2018b). Both frameworks organize mechanism-relevant data in terms of a series of casual events using techniques from acyclic graph theory. The AOP addresses the pharmacodynamic changes caused by chemicals in biological systems and is required by the move from empirical findings of *in vivo* models to approaches that are based on findings of specific mechanisms generated by *in vitro* and *in silico* techniques. The AEP can be viewed as the extension of Conceptual Site Model (CSM), that is used to organize events that occur during manufacture, release, fate, and transport portions of the continuum (Suter, 1999). The AEP includes the exposure to, and the intake of, a chemical and the absorption, distribution, metabolism and excretion (ADME) of the chemical in the individual. Together the AEP and AOP frameworks cover mechanistic data for events that occur over the entire source-to-outcome continuum. The combination of the AOP and AEP have been shown to provide a basis for organizing mechanistic data to support tasks such as the systematic review of data and a workflow for hazard and risk assessments (Jarabek and Hines, 2019).

This paper proposes a new framework for collecting, organizing, and using the mechanistic data generated by NAMs to better understand the effects that can be expected to occur from exposures to chemical mixtures. This new framework seeks to address chemical interactions that occurs over the entire source-to-outcome continuum by using concepts from the AEP and AOP. The paper begins by briefly reviewing existing systems for categorizing chemical interactions. This is followed by a presentation of the two elements that make up the new framework. The use of the new framework for organization and storage including the creation of a semantic triple for chemical interactions is then discussed. An example application of the new framework to a specific type of chemical interaction is provided. Finally, the new framework is used to provide mechanism-based perspectives on the constructs of historical component-based approaches to mixture toxicity.

## 2. Existing approaches for categorizing chemical interactions

The following is a description of the terms and concepts that appear most frequently in publications on mixture toxicology (Bliss, 1939; Finney, 1942; Plackett and Hewlett, 1963; Kodell and Pounds, 1991; Könemann and Pieters, 1996; Hernández et al., 2013; Hernández et al., 2017; Heys et al., 2016). Historically, efforts for classifying joint chemical toxicity have focused on coexposures to chemicals that have a common AO, with less attention given to coexposures where only one (or neither) chemical separately causes an AO of interest.

**Fig. 1.**
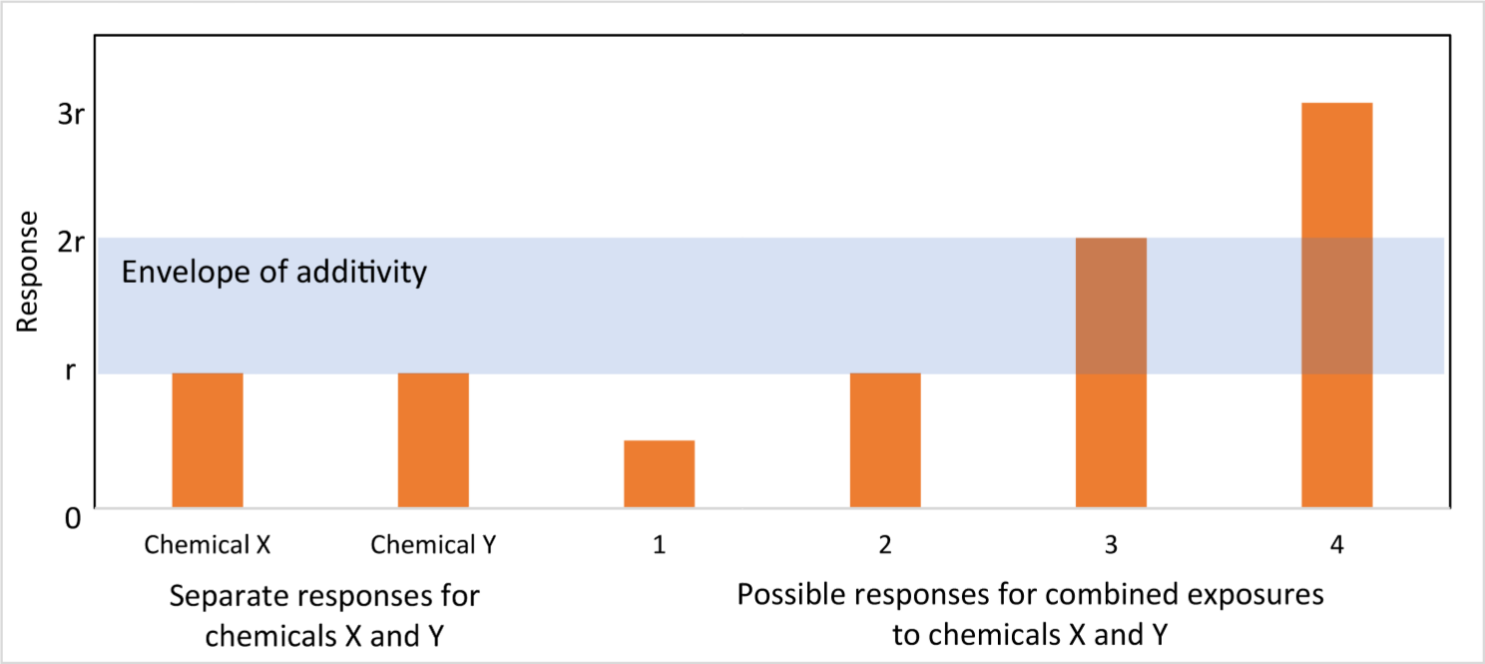
Possible outcomes of empirical testing of two chemicals that cause a common AO as described by Kodell and Pounds (1991). 1: Chemicals X and Y display antagonism, 2: Chemicals X and Y display a response consistent with response additivity where there is a positive correlation in tolerance (same animals sensitive to one chemical are sensitive to the second), 3: Chemicals X and Y display a response consistent with either dose additivity or response additivity when tolerances are negatively correlated (different test animals are affected by the different chemicals), and 4: Chemicals X and Y display synergy.

At different times, and operating from different perspectives, multiple sets of categories and terms have been proposed for interactions of chemicals with common AOs (Kodell and Pounds, 1991; Könemann and Pieters, 1996). Categorization of joint chemical toxicity has typically been based on a comparison of the results of *in vivo* models of the effects of chemicals measured independently and in combination. The system of categories envisions that two chemicals X and Y are given to an *in vivo* model separately. Doses of X and Y are determined which cause a response of r, where r is sufficiently small that the dose response curve can be assumed to be linear. These doses are given concurrently to the same model and the result is used to define the category of the response (Fig. 1).

Responses between a range of r and 2r are taken as evidence that the chemicals may not be modifying each other’s mechanism of action (noninteracting) but simply adding to one another’s effects and are said to be within the envelope of additivity (Kodell and Pounds, 1991). Responses that occur outside of this range are taken as evidence of an interaction between the two chemicals. Responses below r are termed antagonistic interactions and responses above 2r are termed synergistic interactions. For responses within the range of r to 2r, the responses may be dose additive or response additive. In the case of response additivity, coadministration of chemicals X and Y at the highest doses that separately cause a response of zero will not cause a response. In contrast, chemical pairs that follow dose additivity models are expected to cause a response because the combined doses would be sufficient to cause an effect. Finally, instances where chemical X, or chemicals X and Y, do not cause a specific AO when administered separately but do in combination are considered to have demonstrated an interaction.

The existing system has a number of limitations. First, the categories are not defined in terms of any specific mechanism but rather on empirical results. Thus, mechanistic data generated by NAMs do not necessarily indicate the assignment of a chemical interaction into any of the existing categories. Second, empirical findings are specific to the doses tested, the laboratory animal used, and the experimental conditions. Such findings require some sort of model to establish the findings’ relevance for coexposures that occur at lower dose levels, at different ratios, over different durations, or in different species. Such models are difficult to create using only first principles and empirical findings of joint effects. Third, assignment of the interaction between two chemicals to one of the categories can be challenging in practice. Performing *in vivo* studies with limited numbers of animals result in dose response data that lack the statistical power to discriminate between additivity and synergy or additivity and antagonism, especially when the chemicals in a mixture have non-linear dose responses (Kodell and Pounds, 1991; Könemann and Pieters 1996; Hertzberg and MacDonell, 2002). In addition, empirical measurement of response in animal studies may reflect multiple types of interactions operating by different mechanisms at different doses of a mixture (Cassee et al., 1996).

Recently similar approaches have been applied to *in vitro* models of biological activity (Blackwell et al., 2018; Orbach et al., 2018). Studies of interactions that use *in vitro* models of biological activity may include more dose groups and replications and thus have a greater statistical power than *in vivo* studies; however, such assays only measure a single KE or at most a portion of the tested chemicals’ AOPs.

Perhaps most importantly the current system is limited to interactions that occur in the portions of the source-to-outcome continuum that can be studied using *in vivo* and *in vitro* models. These portions begin with the dose given to the test system and end with the AO of the test system. It excludes interactions between chemicals that occur at points between the source of exposure and the external exposure as well as the interactions that occur on the population or ecosystem levels. Interactions between chemicals are known to occur in the transport and transformation processes of chemicals (Spurgeon et al., 2010). For example, chemical X could compete with chemical Y for the transformation processes in media such as air, water, or soil. This competition would increase the amount of chemical Y available to reach a receptor and ultimately cause an effect (Hines et al., 2019). These interactions are characterized in the AEPs of the chemicals (Price and Leonard, 2019).

## 3. A mechanism-based framework for organizing data on chemical interactions

The proposed framework is created by two actions. The first action is to redefine the traditional concepts of chemical interaction and noninteraction in mixture toxicology. The second action is to use the combination of the AEP and AOP frameworks as a basis for a taxonomy of the mechanisms that determine joint toxicity (Price and Leonard, 2019).

### 3.1. Redefining the terms “interaction” and “noninteraction” in mixture toxicology

As discussed in the prior section, most toxicologists have defined chemical “interactions” as observations of joint effects that cannot be explained by an assumption of dose or response additivity. In the proposed framework, interaction is more broadly defined as “*the ability of one chemical (X) to cause a change in the source-to-outcome continuum of a second chemical (Y) for a defined AO*.” This definition of interaction is not novel. It has been used by some researchers (Binderup et al. 2003; Bopp et al., 2019). This definition parallels the basic concept of toxicity presented in the National Academies of Science report *Toxicity in the 21st Century* (NRC, 2007) and the AOP framework (Ankley et al. 2010; Villeneuve et al., 2018). In these documents, toxicity is defined as a perturbation of an existing biological system beyond its normal range by a chemical. In the chemical-interaction framework, a chemical interaction is defined as a perturbation of the existing source-to-outcome continuum of chemical Y by chemical X.

As discussed in the prior section, many toxicologists have defined “noninteraction” as chemical mixtures that follow either a dose or response addition model (Kodell and Pounds 1991; Hernández et al., 2013). In the proposed framework, “noninteraction” between chemicals X and Y is defined as “*the lack of the ability of X to change the source-to-outcome of Y at any dose of X.”* Under this definition chemical X cannot cause the AO of Y nor can it change the relationship between a release of Y, or a dose of Y, and an AO. Strictly speaking no two chemicals are noninteracting at all doses (any chemical X when given at sufficiently toxic doses, will change the response of chemical Y by killing the model organisms prior to the display of the AO); however, if the maximum tolerated dose of chemical X does not change the ability of Y to cause the AO, then chemical X can be said to be noninteracting with respect to Y. This definition is similar to the concept of “no apparent influence” as proposed by EPA (USEPA, 2000).

In the proposed chemical-interaction framework, if chemicals X and Y cause a common AO and follow dose addition or response addition models they are considered to be interacting. As discussed below, the possible mechanisms that could be associated with either dose or response addition can be defined using the topology of AOP networks (Villeneuve et al., 2018; Nelms et al., 2018) and can be assigned to specific categories of interactions (Price and Leonard, 2019).

A major implication in the new definition of interaction between two chemicals is that each chemical plays a different role in the interaction. Historically, the focus of *in vivo* studies was to determine the nature of the joint action and not necessarily the specific roles for each chemical.

$$\text{Dose}\;\text{of}\;\text{X}\to {\text{Outcome}}_{\text{X}}\;\text{Dose}\;\text{of}\;Y\to {\text{Outcome}}_{\text{Y}}\;\text{Dose}\;\text{of}\;X\;\text{and}\;Y\to {\text{Outcome}}_{\text{X+Y}}$$

In the proposed framework, data on an interaction is stored in terms of chemical X interacting with chemical Y’s source-to-outcome continuum and changes in the response of the AO to a given release, or dose, of Y. Such a definition is directional with chemical X changing the AEP or AOP of Y.

$$\text{Release}\;\text{of}\;Y\overset{\underset{↓}{X}}{\to }{\text{Outcome}}_{\text{Y}}$$

This approach can be used for pairs where both chemicals separately cause an AO of interest, where only one of the two chemicals separately causes the AO, and when neither causes the AO separately. When two chemicals both cause an AO, however, it is often useful to capture differences in the roles that each chemical plays in any interaction. To capture such interactions a database would store information on the interaction as two entries, first as the effect of X on the ability of Y to cause an AO and second as the effect of Y on the ability of X to cause an AO.

### 3.2. Using the AEP-AOP as the basis for a taxonomy of interactions

The second component of the proposed framework is a previously published taxonomy of chemical interactions (Price and Leonard, 2019). The following is a brief summary of that taxonomy. The reader is encouraged to read the original publication for additional information.

**Fig. 2.**
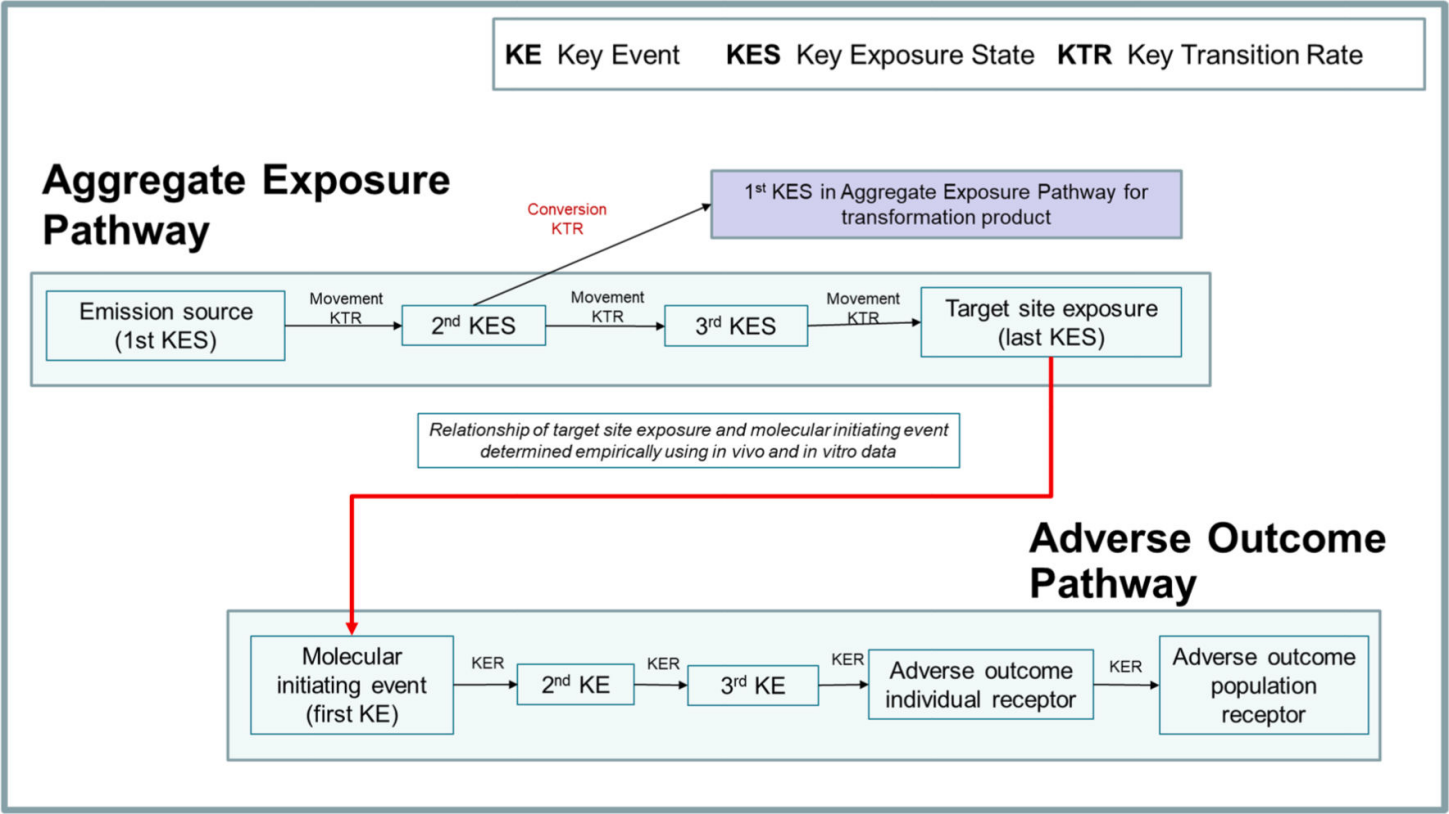
Using a combination of the AOP and AEP to characterize the causal events in the source-to-outcome continuum (taken from Price and Leonard, 2019).

The taxonomy addresses all chemical interactions that occur over the source-to-outcome continuum of a chemical risk assessment. This source-to-outcome continuum is defined using a combination of the AEP and AOP (Fig. 2).

**Fig. 3.**
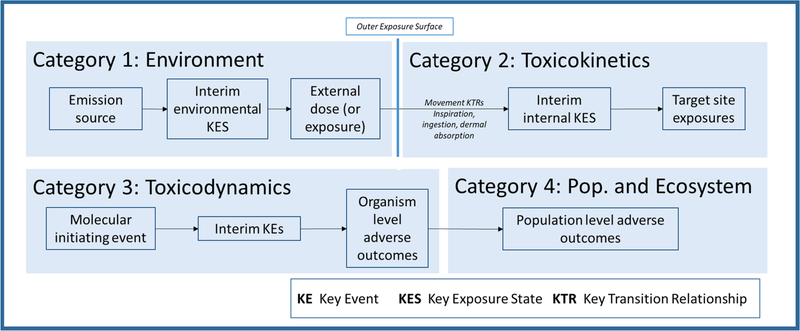
Regions of the source-to-outcome continuum that define the four top level categories of the proposed taxonomy (taken from Price and Leonard, 2019).

The location of the interaction on the continuum is proposed as a criterion for organizing chemical interactions (Fig. 3). The continuum is divided into four contiguous and non-overlapping regions that cover the entire continuum. The interactions that occur in a region are assigned to the top tier category of the taxonomy that corresponds to the region. The resulting system of four categories is exhaustive (all interactions will fall into one of the categories) and mutually exclusive (an interaction will fall into only one category). These four top categories are divided into subcategories defined using concepts derived from the AOP and AEP (Table 1). Table 1 also presents an example interaction for each category and subcategory.

The taxonomy as presented in the 2019 publication (Price and Leonard, 2019) is meant to be an initial attempt. Future versions of the taxonomy would be expected to add additional tiers that categorize the specific mechanisms and biological processes involved in two chemicals’ interactions. It is also possible that the two proposed tiers may be revised to reflect future changes in the AEP and or the AOP. Finally, the taxonomy is based on the interactions between two chemicals. More complex interactions involving three or more chemicals are assumed to be captured by the interactions between the individual pairs of chemicals that make up the group. This assumption will be a topic for future research

## 4. Using the proposed framework to organize data on interactions

### 4.1. Directed interactions as the basis for a semantic triple

Multiple groups and organizations are working to manage mechanistic data on chemical toxicity. AOPs are being stored in an international AOP knowledgebase (Organisation for Economic Co-operation and Development, 2013; Society for the Advancement of Adverse Outcome Pathways, 2013). Ontologies have been proposed for AOPs (Ives et al., 2017; Burgoon, 2017) and are used to enhance the knowledgebase for various purposes. In addition, ontology practitioners are beginning to look at graph databases to achieve these aims.

**Fig. 4.**
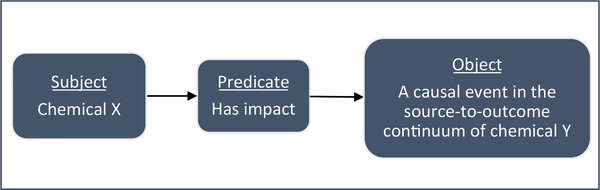
Directed interaction of two chemicals presented as a semantic triple.

The proposed framework may be useful in the design of a knowledgebase for data on chemical interactions. The creation of a knowledgebase requires actions such as standardizing vocabularies, creating taxonomies, and establishing data formats in order to enhance data utility. Such knowledgebases should be consistent with FAIR principles and meet the needs for reproducibility and rigor (Wilkinson et al., 2019; Waller and Miller, 2016). When creating an ontology for an area of study it is essential to identify the structure of the essential concepts that define that area of study. We propose that the definition of directed interaction can serve as the essential concept for chemical interactions. In addition, the structure of the directed interaction can be expressed as a semantic triple that could support a semantic Resource Development Framework (RDF) for data on chemical mixtures (Fig. 4). RDFs are databases are designed facilitate the web-based searches for data. Semantic triples encode the relationships between concepts in an ontology, where a concept consists of a subject, a predicate and an object – similar to English grammar (Ives et al. 2017).

The object of the triple is an event in the source-to-outcome continuum of Y. The event is defined as a KTR in the AEP, or as a KER in the AOP that is changed as a result of the effects of chemical X.

The predicate of the semantic triple serves as a “bridging” function that connects the subject to the object. In the proposed triple, we use the generic language “has impact” to mean that the subject has an “effect or consequence of an event or condition” on the object. This is consistent with the National Cancer Institute Thesaurus definition for “impact” (http://ncicb.nci.nih.gov/xml/owl/EVS/Thesaurus.owl#C122929, viewable at https://bioportal.bioontology.org/ontologies/NCIT/?p=classes&conceptid=http%3A%2F%2Fncicb.nci.nih.gov%2Fxml%2Fowl%2FEVS%2FThesaurus.owl%23C122929). As discussed in the paragraphs immediately following, the impacts are different for events that occur in different portions of the continuum. In the future we could see more exact language being used in the predicate to capture these differences, or an even more specific predicate from another appropriate ontology, being used in lieu of the generic “has impact”.

The impacts for the processes addressed by the AEP (Categories 1 and 2) can be grouped using the changes in the KTRs associated with the subcategories of Categories 1 and 2. The impacts that occur in the portion of the continuum covered by the AOP (Categories 3 and 4) involve a common AOP network that defines the interaction. AOP networks are chemically agnostic. As a result, the interactions are defined in terms of the MIEs that chemicals X and Y cause in the AOP network. This suggests that impacts of these interactions are defined by the following: (1) whether X and Y cause the same MIE or if they cause different MIEs, and (2) the topology of the downstream AOP network of the MIEs. Chemical pairs that have the same MIE fall into Subcategory 3A. These impacts are determined by the nature of the relationships between the Target Site Exposures (TSEs) of X and Y to the common MIE. Pairs of chemicals that cause different MIEs fall into Subcategories 3B and 3C. As defined by Villeneuve et al. (2018) both 3B and 3C interactions occur on apical convergent networks (separate MIEs leading to a common AO). The 3B interactions have AOs that are downstream of an Initial Common Key Event (ICKE) that in turn is downstream of the MIEs of X and Y. The KER that relates the effects of X to the ICKE defines the impact for a 3B interaction. The 3C interactions have the AOPs that are downstream of the MIEs and KEs of X and Y meeting at a common AO. The final KER of X’s AOP defines the impact of X.

The subject of the triple is chemical X. Chemical X acts to change the source-to-outcome of chemical Y. The subject can be linked to metadata that helps define the interaction. This metadata includes the properties of chemical X that are relevant to the interaction. These properties can be divided into three groups physical properties, chemical properties, and toxicological properties. The impacts of chemical X (but not necessarily chemical X itself), must be present at the location of the interaction (defined as the location of the relevant KES or KE) and a point in time that is concurrent with the presence of chemical Y (or its effects) at the location. As a result, the data on chemical X should include information on the chemical X’s AEP and AOP up to the point where chemical X’s effect on chemical Y occurs.

### 4.2. Using the framework to define a chemical interaction network

**Fig. 5.**
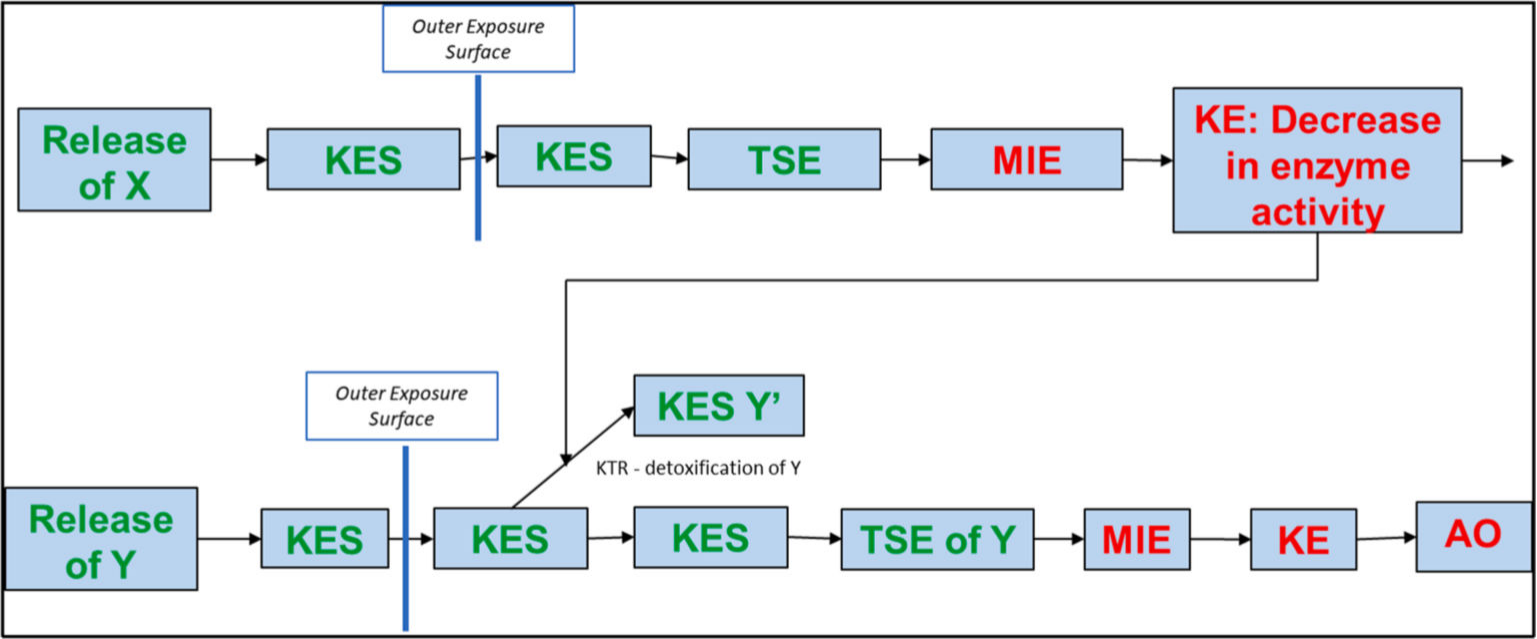
An AEP-AOP network for two chemicals with an interaction falling into Subcategory 2B: chemical X modifies the metabolism of chemical Y decreasing the detoxification of chemical Y and resulting in a synergistic interaction.

Fig. 5 presents an example of how the triple can be used to describe a common type of chemical interaction. In this interaction chemical X is the object. It reaches an organism (described in the AEP of chemical X) and causes a MIE that leads to a change in enzyme activity in the organism (described as a key event in the AOP of chemical X). An impact of X is the decreased enzyme activity in the organism. The object is a conversion KTR in the AEP of chemical Y that is determined by the affected enzyme. The decrease in the activity of the enzyme decreases the removal of Y (detoxification) and results in an increase in the TSE of Y. The increase in the TSE results in an increase in the response rate for the AO associated with the release of Y (synergy). An example of an interaction that follows this example would be the effect of grapefruit juice consumption on drug metabolism (Lilja et al., 2004).

While AOP networks have been used to characterize the interactions of chemicals (Villeneuve et al., 2018; Knapen et al., 2015) the AOP networks only capture the chemical interactions that occur on one portion of the source-to-outcome continuum (toxicodynamic interactions). The AEP-AOP network links the AEP and AOP of chemical X to a process in the AEP of chemical Y. This linking of an AOP of chemical X to an AEP of Y is required to characterize many interactions in Category 2.

### 4.3. Using the framework to provide a mechanism-based perspective of existing constructs in component-based approaches in mixture toxicology

As discussed above, constructs in component-based mixture toxicology (categories and implications of those categories) have been based on empirical measurements of separate and joint toxicity (Boobis et al., 2011; Kodell and Pounds, 1991). In this section the framework is used to link specific mechanisms to the existing constructs of component-based mixture toxicology. This is not to say that mechanistic concepts have not been discussed in the literature (Ariens et al., 1976; Kodell and Pounds, 1991; Spurgeon et al., 2010; Bopp et al., 2019); however, such discussions have been limited by the absence of a coherent mechanism-based framework for interactions. Unlike the redefinition of the terms “interaction” and “noninteraction” proposed above, the goal in this section is not to change the definitions of the constructs, but to provide alternative mechanism-based definitions that may serve as bridges between the constructs and mechanistic findings.

#### 4.3.1. Interaction thresholds

Interaction thresholds occur when chemical X has a specific type of interaction at one dose but not at a lower dose. Thresholds of interactions have been observed in empirical measurements of joint response (Hamm et al, 2005; Yeatts et al. 2010) and have been described using PBPK models (El-Masri et al. 2004). One of the mechanisms by which such interaction thresholds occur is when chemical X causes its impact by means of its toxicological effects. Several of the interaction categories are based on the impact of the toxicological properties of chemical X on the source-to-outcome of chemical Y. These including certain interactions in Subcategories 1A, 1B, 2A, and 2B and all interactions in Subcategories 3B, 3C, 4A and 4B. In these interactions, chemical X must reach an organism and cause a MIE leading to KEs and AOs in its own AOP. Thresholds would be expected for these interactions. For example, chemical X would only affect chemical Y in a 3B interaction when the TSE of X was sufficiently large to cause the KE immediately prior to the ICKE for Y. Such an exposure may be lower than the level necessary to cause the AO for X and will be the same or higher than the dose causing the MIE. Below this dose X would not affect the dose response of Y. Chemical X would only affect chemical Y in 3C interactions when the TSE of X was sufficiently large to independently cause the AO. Thresholds could also occur for interactions where the chemical and physical properties of chemical X are the cause of the interaction. For example, pH-related interactions where chemical X was an acid or base and the KES contained buffers would display threshold-type behaviors.

#### 4.3.2. Dose addition

The dose addition model assumes that two chemicals act as if they are simple dilutions of a single chemical (USEPA, 2003; IGHRC, 2009), causing a common AO, having the same sites of primary action, and the same mode of action at the site (Kodell and Pounds, 1991). An important implication of dose additivity is that no interaction threshold exists for the chemicals that follow dose addition (Könemann and Pieters, 1996). Under a dose additivity model, contributions from large numbers of small doses of chemicals may pose a risk. As a result, a mixture with an ED_10_ could be created by taking half of the ED_10_ of two chemical or 1/100 of the ED_10_ of one hundred chemicals. The reason for this is that the interaction occurs at a single MIE and is determined by the combined TSEs of the chemicals.

Under the characteristics of dose addition provided above, interacting with a common site (e.g., within a tissue or cell) is a necessary, but not a sufficient, finding for dose additivity. Interactions that involve a common site may occur by different AOPs. For example, in a receptor-mediated effect where the receptor was located in a specific type of cell, chemical Y could bind to the receptor leading to the AO and chemical X could cause toxicity to the cell containing the receptor. Such an interaction has a common site but would not be dose additive at doses of X that were below the threshold of the MIE that led to chemical X’s cytotoxicity. The potential for dose additivity only occurs in the AOP framework when two chemicals affect a common MIE (Nelms et al., 2018). Such interactions would fall under Subcategory 3A.

Even with a common MIE, however, the joint response need not follow a dose additivity model. To return to the above example, in a receptor-mediated MIE, receptor binding for different chemicals can vary from weakly binding low activity compounds to chemicals that irreversibly block a receptor. The effects of combined exposure of such chemicals would not necessarily follow dose additivity at all doses. In this case a quantitative AOP (qAOP) that included modeling of receptor binding would be necessary to predict the combined effects.

Based upon these findings, the following mechanism-based definition of dose addition is proposed:

Dose addition occurs between two chemicals (X and Y) when a prior, or concurrent, exposure to chemical X causes an increase in the intensity or duration of the MIE in response to a given release of Y from a source (or a given dose of Y) by acting as if it was a concurrent toxicity-weighted TSE of Y.

#### 4.3.3. Response addition

In response addition models a chemical component of a mixture will not contribute to a mixture’s toxicity unless it is present at a sufficient dose to cause a response independently (Kodell and Pounds, 1991). When expressed in terms of an AOP network, such interactions would occur when two chemicals have separate MIEs and KEs but converge to a common AO (Nelms et al., 2018). As a result, addition interactions would fall under Subcategory 3C of the taxonomy and have interaction thresholds.

Based upon these findings the following mechanism-based definition of response addition is proposed:

Response addition occurs between two chemicals (X and Y) when a prior, or concurrent, exposure to chemical X causes an AO in an exposed population and changes the response to a given release of Y from a source (or a given dose of Y) by reducing the number of individuals where the AO has not occurred.

#### 4.3.4. Combined toxicity models for Subcategory 3B interactions

While 3A and 3C interaction map to dose additivity and response additivity, Subcategory 3B interactions do not fit well into either type of additivity. As discussed above, interactions that fall under 3B (having a common KE in an AOP network) form convergent AOP networks. The nature of the joint toxicity for these interactions is determined by the KERs that directly link to the ICKE of the network and connect back to the MIEs of chemicals X and Y (Villeneuve et al., 2018; Conley et al., 2018).

- If the impact of the KER in chemical X’s AOP that links to the ICKE is the same as the KER in chemical Y’s AOP that links to the ICKE, the impacts would be response additive. For example, if the relevant KERs of both X and Y result in the death of a specific type of cell.
- If the impact of the KER in chemical X’s AOP that links to the ICKE is in the same direction as the KER in chemical Y’s AOP that links to the ICKE but differs in mechanism, the impacts could differ from additivity and could be supra-additive (synergistic) or sub-additive (partial additivity). For example, if the KER for Y was to cause toxicity in a specific cell and X inhibits the replacement of the cell, the combined effect would be positive (X would make Y more toxic) but the response need not follow dose additivity (e.g., a dose of X that prevents cell replacement might make a long-term dossing regime of Y that separately caused minimal cytotoxicity highly toxic to an organism).
- If the impact of the KER in chemical X’s AOP that links to the ICKE is in the opposite direction to the KER in chemical Y’s AOP that links to the ICKE, the impact would be antagonistic (e.g., Y suppresses an enzyme’s activity and X increases the activity). The form that the antagonism would take would depend on the quantitative relationships of the KERs for X and Y that link to the ICKE.

Because of the dependence on the KERs for chemicals of X and Y that link to the ICKE, it is not possible to predict the nature of the joint response without the construction of a qAOP network for the two chemicals. One characteristic of all 3B interactions, however, is that they will have interaction thresholds. The effects of X would add to the effects of Y only when the TSE of X was sufficiently large to cause the KE immediately prior the ICKE in chemical Y’s AOP.

Nelms et al. (2018) has proposed 3B interactions could be modeled using dose addition. Such an approach would be conservative since it would not consider the threshold in the impacts of X. However, it may not be conservative if the interaction was supra-additive. This area undoubtedly will be the subject of ongoing research.

#### 4.3.5. Synergy and antagonism

The definitions of synergy and antagonism have a long and complex history. As discussed above, many researchers defined synergy and antagonism as deviations from the responses that would be expected to occur under either dose and response additivity (Kodell and Pounds 1991; Hernández et al., 2017; Boobis et al. 2011). Such a definition only requires empirical testing for a determination and not a specific mechanism. Researchers, however, have discussed the mechanistic bases for synergy and antagonism. Ariens et al. (1976) suggested that synergy could be divided into interactions in the kinetic and dynamic phases of toxicity. Heys et al. (2016) and Bopp et al. (2019), discuss the mechanisms involved with kinetic interaction including changes in metabolism that decrease or increase the internal dose of the chemical or its active metabolite resulting from a certain administered dose.

Within the proposed framework, synergy and antagonism are viewed as follows. In the portion of the source-to-outcome continuum covered by the AEP framework, a molecule of a chemical released from a source may or may not reach an organism. If it reaches an organism it may reach the target site of the MIE or it may be excreted or metabolized. If the chemical requires activation, the molecule may not be activated, or if activated, the active compound may not reach the target site. Kinetic synergy occurs when a prior, or concurrent, exposure of chemical X increases the portion of the release of chemical Y that reaches the target site in the form of Y or its active metabolite (Heys et al., 2016; Bopp et al., 2019). Kinetic antagonism occurs when a prior, or concurrent, exposure to X decreases the portion of the release of Y that reaches the target site in the form of Y or its active metabolite.

Dynamic interactions are investigated using AOP networks (Knapen et al., 2015; Burgoon et al., 2017; Nelms et al., 2018; Villeneuve et al., 2018; Perkins et al., 2019). An organism may have, or in response to a chemical’s perturbation may develop, excess capacity or redundancies in its systems that reduce the potential for the occurrence of the AO given a MIE of a specific intensity and duration. When chemical X suppresses one or more of these functions, it changes the quantitative relationship between the MIE and the AOP for Y and results in dynamic synergy. When a chemical X enhances one or more of these functions in the AOP of chemical Y it results in dynamic antagonism. The AEP-AOP framework and the taxonomy provides a basis for defining synergy and antagonism. Kinetic synergy could occur as a result of interactions in all the subcategories of Categories 1 and 2. Dynamic synergy can occur as a result of interactions in subcategories 3A, 3B, 4A and 4B.

Based upon the above, the definitions of synergy and antagonism can be stated as follows:

Synergy occurs between two chemicals, X and Y, when a prior, or concurrent, exposure to chemical X causes an increase in the response to a release of Y from a source by (1) increasing the ratio of the amount of Y released by a source and the TSE for Y, or its active metabolite (kinetic synergy) or (2) by increasing the probability that a MIE of a given intensity and duration will result in the AO (dynamic synergy).

Antagonism occurs between two chemicals, X and Y, when a prior or concurrent exposure to chemical X causes a decrease in the response to a release of Y from a source by (1) decreasing the ratio of the amount of Y released by a source and the TSE for Y, or its active metabolite (kinetic antagonism) or (2) by decreasing the probability that an MIE of a given intensity and duration will result in the AO (dynamic antagonism).

As discussed above, interactions are a function of the properties of chemical X (physical, chemical, or toxicological). The interaction can be expected to have thresholds when synergy and antagonism occur as a result of the impact of the toxicological and certain physical and chemical effects of chemical X.

## 5. Discussion

This paper presents initial thoughts for the creation of a framework for organizing, storing, and using data on chemical interactions in the assessment of risks from combined exposures. The work is based on the AEP, the AOP, a redefinition of “interaction” that is directional and mechanistic, and the use of an existing taxonomy of chemical interactions. There are a number of benefits of the resulting chemical-interaction framework. The chemical-interaction framework addresses chemical interactions that occur at any point in the source-to-response continuum and can integrate both AOPs that occur at the individual and population levels. The categories and subcategories of the taxonomy are mutually exclusive and the hierarchical relationships between a category and its subcategories are objectively defined. The semantic triple that flows from the framework could provide a basis for systematically extracting, organizing, and storing information on chemical interactions.

Unlike the AOP, the proposed chemical interaction framework is not chemically agnostic. Interactions are a function of the specific chemicals and cannot be defined in isolation of the chemicals involved. The proposed framework uses the AEP to address interactions that occur in the release (emissions), transport, conversion, exposure and dosimetry, and TSE portions of the continuum and the AEP is not chemically agnostic. The approach can take advantage of the chemically agnostic AOP networks by specifying the MIEs that are affected by the interacting chemicals.

Organizing chemicals’ interactions based on the location of the interaction on the source-to-outcome continuum will identify groups of chemicals involved with specific types of interactions. Nelms et al., (2018) proposed that the identification of chemicals that impact a common MIE (and thus fall into Subcategory 3A) could provide a basis for read-across models that predict untested chemicals’ potential to cause the MIE. Chemical predicted to cause a MIE would form a grouping of chemicals likely to cause 3A interactions with each other. Such groupings would also provide the ability to predict 3B interactions. Consider two groups affecting different MIEs on a convergent AOP network (leading to the same AO). Where a chemical from each of the two groups reaches an individual there would be a potential for a 3B interaction to occur. A finding of such a combined exposure could then trigger an investigation of whether the doses of the two chemicals are sufficiently large to cause an interaction.

The chemical-interaction framework is anticipatory. Current data on chemical interactions are limited to a small number of chemical combinations and many of the existing studies do not report all the data necessary to use the proposed framework. The value of the framework is both to begin the process of creating databases of interactions that can be used to organize data as it becomes available and to identify the data that should be captured in future studies of chemical interactions.

Finally, the framework presented in this work, like the taxonomy presented in Price and Leonard (2019), is an initial step in organizing data on chemical interactions. The framework and taxonomy are likely to be expanded and modified in the future. One area of research will be to develop approaches where two or more chemicals have MIEs on more complex networks or where chemicals cause multiple MIEs at different TSEs. Such groups of chemicals may generate multiple types of interactions that fall into different subcategories. In the near term, AEP-AOP networks based on the framework can help guide the development of workflows for both the experimental designs and evaluations conducted in mixture risk assessments.

## Figures and Tables

**Table 1:** Categories and subcategories in the AEP-AOP based taxonomy of chemical interactions from Price and Leonard (2019) and example interactions.

Category	Example	Reference

**Category 1.** Interactions occurring during environmental exposure processes, including emissions, transport and transformation, and exposure processes. Chemical X may interact with a chemical Y by either:		
1A. Influencing the movement of chemical Y between environmental KESs	Effects of acids on the mobility of metals in soils and aquatic systems	Spurgeon et al. (2010)
1B. Changing the conversion rate of chemical Y in an environmental KES	Reducing the conversion of ammonia to nitrate in soil by dicyandiamide	Rose et al. (2018)
1C. Creating a new conversion KTR that involves chemicals X and Y in an environmental KES	Photochemical reaction of nitrogen oxide and methane to produce formaldehyde in the atmosphere	Luecken et al. (2018)
**Category 2.** Interactions during the toxicokinetic processes. Chemical X may interact with chemical Y by either:		
2A. Influencing the movement of chemical X between KESs in an organism	Increased of dermal absorption of disinfection-by-products by sodium lauryl sulfate	Trabaris et al. (2012)
2B. Changing the conversion rate of chemical X in an organism’s KES	Ethanol’s ability to inhibit the metabolism of methanol by competitive inhibition of alcohol dehydrogenase	Tephly (1991)
2C. Creating a new conversion KTR that involves chemicals X and Y in an organism’s KES	The ability of melamine and cyanuric acid to form insoluble chemical complexes in the kidney leading to nephrotoxicity	Puschner et al. (2007), Dorne et al. (2013)
**Category 3.** Chemical Interactions that involve chemicals with MIEs on a common AOP network		
3A. Chemicals X and Y have one or more common MIEs	Thyroid hormone disruption caused by sodium-iodide symporter (NIS) inhibitors such as perchlorate, thiocyanate, and nitrates.	Hines et al. (2019)
3B. Chemicals X and Y have different MIEs but have one or more common intermediate KEs	Stimulation of estrogen receptor by bisphenol A and inhibition of androgen receptor by diethyl hexyl phthalate both leading to common KEs and a common AO of reduced fertility	De Falco et al. (2015)
3C. Chemicals X and Y have different MIEs, different intermediate KEs, and a common AO	Pulmonary fibrosis that is caused by nickel oxide nanoparticles and cigarette smoke	Bai et al. (2018) and Checa et al. (2016)
**Category 4.** Interactions leading to an adverse outcome in a population due to population- or ecosystem-mediated interactions.		
4A. Chemicals X and Y have different MIEs for AOs that occur in different portions of a receptor population	Flubenzuron a larvicide for juvenile sea lice and pyrethroids are pesticides that affect adult sea lice	Van Geest et al. (2014)
4B. Chemicals X and Y have different AOs in different species in an ecosystem, but the AOs lead to a joint effect in a receptor population	Turbufos causes direct toxicity cladocerans while atrazine reduces the levels of food (algae) for the planktonic animals	Choung et al. (2013)

## Abbreviations:

ADME: absorption, distribution, metabolism and elimination
AEP: Aggregate Exposure Pathway
AO: Adverse Outcome
AOP: Adverse Outcome Pathway
CSM: Conceptual Site Model
ICKE: Initial Common Key Event
KE: Key Event
KER: Key Event Relationship
KES: Key Exposure State
KTR: Key Transition Rate
MIE: Molecular Initiating Event
NAM: New Approach Methodology
RDF: Resource Description Framework
TSE: Target Site Exposure
qAOP: quantitative Adverse Outcome Pathway
